# Unhappiness and mortality: evidence from a middle-income Southeast Asian setting

**DOI:** 10.1186/1751-0759-8-18

**Published:** 2014-08-07

**Authors:** Vasoontara Yiengprugsawan, Sam-ang Seubsman, Adrian C Sleigh

**Affiliations:** 1 National Centre for Epidemiology and Population Health, College of Medicine, Biology and Environment, Research School of Population Health, The Australian National University, Building 62, Mills Rd, Canberra, Acton 2601, Australia; 2 School of Human Ecology, Sukhothai Thammathirat Open University, Nonthaburi, Thailand

**Keywords:** Happiness, Mortality, Psychological wellbeing, Cohort study, Thailand

## Abstract

**Background:**

A relationship between happiness and mortality might seem obvious, but outside of affluent settings in developed countries there is almost no actual evidence that this is so.

**Findings:**

We report our findings on happiness and mortality in Buddhist Southeast Asia. Our data are derived from a prospective nationwide cohort study of 60,569 Thai adults reporting in 2009 and followed up for all-cause mortality over the next four years (296 deaths). We also gathered data on a wide array of covariates and included these in the final model of the unhappiness-mortality effect. All final effect estimates were mutually adjusted odds ratios (AOR) and cohort members who reported being happy ‘little’ or ‘none of the time’ in 2009 were more likely to die (AOR 2.60, 95% Confidence Interval 1.17-5.80). Other significant covariates include being female (<40 years AOR 0.66, ≥40 years AOR 0.57), unmarried (AOR 1.64) and current smokers (AOR 2.45).

**Conclusion:**

Our study provides empirical evidence that the epidemiological effect of happiness is not confined to affluent Western countries, but it also increases the probability of staying alive in a middle-income Asian country.

## Findings

When the 14^th^ Dalai Lama accepted his Nobel Prize in 1989, he observed that “…we are all basically the same… we all seek happiness and try to avoid suffering.” These connections were recognised in 1972 when King Jigme Wangchuck of Bhutan, drawing on Buddhist principles, famously focused development goals on boosting the Gross National Happiness [1]. A high level of happiness and minimal suffering has shown to interact to produce health and wellbeing [2-4] and Western studies report that subjective wellbeing, life satisfaction, and happiness not only associate with good health [5,6] but also protect against premature mortality [7-9]. However, almost nothing has been reported about happiness effects on mortality in other economically poorer or culturally different (non-Western) settings.

In the field of positive psychology, happiness has been investigated from both subjective (hedonic) and psychological (eudaimonic) perspectives [4,10]. For quantitative population health research, self-reported happiness is commonly measured on scales including the World Values Survey and European Values Study, which have been systematically carried out between 1981 and 2007 [11]. Happiness was assessed by asking respondents to indicate how happy they were using four ordinal categories: *very happy*, *rather happy*, *not very happy*, and *not at all happy*. Subsequent studies on happiness have applied similar measures [8,12,13].

We report our study of happiness and mortality in a Buddhist middle-income Southeast Asian country, using a large prospective cohort studying the health-risk transition among 87151 Thai adults. We began this cohort in 2005 with a comprehensive mail-out questionnaire to Open University distance-learning adults residing nationwide. The mean age was 29 years, half were female, half were urban, and most had modest incomes similar to the general Thai population [14,15]. In addition to a wide array of social and health information, we also collected Citizen IDs which were later used to match with national death records.

At the 2009 cohort follow-up (n = 60569), we measured happiness by self-report (five ordinal categories: all, most, some, little, or none of the time). Unhappiness was classified for those reporting being happy ‘little’ or ‘none of the time’. Over the next four years, using the Citizen ID numbers and national vital statistics, we detected 296 deaths among the 60569 cohort members studied in 2009. This 4-year longitudinal analysis focused on unhappiness and its association with mortality. Covariates investigated include sociodemographic characteristics (age-sex categories, age in years, marital status, household monthly income, paid work hours, rural-urban residence), health behaviours (smoking, alcohol drinking, physical activity), health states (self-assessed overall health and doctor diagnosed cardio-metabolic conditions), and social capital (interaction with family, friends, neighbours, and colleagues). These covariates were also controlled for in a recent systematic review of 70 quantitative studies on positive wellbeing and mortality [7].

For our study, using the 2009 cohort follow-up data, we first performed a bivariate analysis of the cross-sectional relationship between covariates and unhappiness (Table 1). Then we conducted a 4-year longitudinal analysis of 2009 unhappiness as a predictor of mortality for the 296 deaths occurring in the period over 2009-2012 (Table 2). For this analysis of the effect of unhappiness on mortality, the odds ratios for each variable in the model are adjusted for the influence for all the covariates listed in Table 1. These Adjusted Odds Ratios (AOR) are presented together with their 95% Confidence Intervals in Table 2.

**Table 1:** Unhappiness and mortality by cohort attributes, Thai Cohort Study

**Cohort attributes**	**% proportion (n = 60569)**	% reported unhappy*		Bivariate odds ratio** [95% confidence intervals] for unhappiness by cohort attributes
		**Alive in 2012**	**Dead (n=296)**	
**Overall cohort in 2009**		5.2 (3068)	8.1 (24)	
**Sociodemographic attributes**				
***Age-sex groups:***				
Males <40 years	24.8	5.3 (1107)	7.6 (9)	Reference
Females <40 years	33.9	5.3 (1554)	10.5 (8)	0.95 [0.86-1.05]
Males ≥40 years	20.6	4.0 (226)	6.3 (5)	0.68 [0.57-0.81]***
Females ≥40 years	20.8	5.1 (181)	4.8 (1)	0.80 [0.66-0.98]***
***Marital status:***				
Married	55.3	4.3 (1282)	5.0 (7)	Reference
Not married	37.9	5.6 (1158)	9.8 (9)	1.35 [1.22-1.50]***
Divorced/widowed	6.8	8.7 (320)	11.8 (4)	2.34 [1.97-2.77]***
***Household monthly income (Baht)*:**				
≤10000	21.8	7.6 (962)	9.0 (6)	1.91 [1.68-2.16]***
10001-20000	24.3	5.0 (709)	8.3 (6)	1.40 [1.20-1.56]***
20001-30000	21.9	4.8 (606)	5.6 (4)	1.40 [1.23-1.61]***
>30000	31.9	3.7 (690)	9.7 (7)	Reference
***Paid work:***				
≤10 hours per week	31.4	5.9 (1062)	4.2 (4)	Reference
10-40 hours per week	33.9	4.5 (870)	5.4 (5)	0.90 [0.80-1.01]
>40 hours per week	34.8	4.9 (973)	15.1 (13)	1.12 [0.99-1.24]
***Residence:***				
Rural	44.0	5.2 (1344)	7.6 (10)	Reference
Urban	56.0	5.1 (1653)	8.4 (13)	0.99 [0.94-1.06]
**Health behaviours**				
***Smoking:***				
Never	76.8	4.9 (2190)	8.2 (14)	Reference
Current	8.9	7.2 (369)	5.2 (3)	1.89 [1.63-2.19]***
Former	14.3	5.2 (434)	8.1 (5)	1.22 [1.08-1.39]***
***Alcohol drinking:***				
None per week	72.9	5.0 (2078)	8.3 (16)	
1-2 sessions per week	19.1	4.9 (534)	9.1 (5)	1.31 [1.16-1.48]***
3+ sessions per week	8.0	6.3 (289)	2.6 (1)	1.60 [1.36-1.88]***
***Physical activity:***				
None per week	22.5	5.7 (1850)	6.2 (9)	Reference
1-2 times per week	33.2	5.3 (1021)	8.1 (6)	0.71 [0.63-0.81]***
3-5 times per week	32.6	4.2 (806)	10.2 (11)	0.41 [0.36-0.47]***
>5 times per week	11.7	5.0 (341)	8.8 (3)	0.37 [0.32-0.44]***
**Health and social attributes**				
***Self-assessed health:***				
Excellent/very good	64.6	3.0 (1159)	4.9 (9)	Reference
Fair	29.9	7.1 (1266)	10.3 (9)	5.7 [5.10-6.33]***
Poor or very poor	5.4	19.4 (627)	23.1 (6)	19.7 [0.42-0.56]***
***Cardio-metabolic conditions:***				
No	81.1	5.1 (2349)	7.5 (14)	Reference
Yes	18.9	5.2 (715)	9.3 (10)	1.13 [1.02-1.26]***
***Social interaction:***				
Every week	17.0	3.0 (306)	1.7 (1)	Reference
1–2 times/month	64.8	4.5 (1645)	8.2 (13)	2.12 [1.55-2.44]***
Rarely/never	18.2	8.8 (903)	15.8 (9)	5.02 [4.28-5.88]***

*Unhappiness is classified in 2009 as those reporting happy ‘little’ or ‘none of the time’.

**Bivariate odds ratios are adjusted for age-sex categories for all variables and estimate effects of unhappiness.

***Statistical significance at p<0.05.

**Table 2:** Self-reported happiness as a predictor of mortality, Thai Cohort Study 2009-2012

**Cohort attributes**	Multivariate adjusted odds ratio* [95% confidence interval] of mortality predictor
Self-reported happiness**	
All of the time	Reference
Most of the time	1.66 [0.88-3.11]
Some of the time	1.69 [0.88-3.23]
Little of the time	**2.60** [1.17-5.80]***
**Sociodemographic attributes**	
***Age-sex groups:***	
Males <40 years	Reference
Females <40 years	**0.66** [0.43-0.99]***
Males ≥40 years	1.34 [0.78-2.31]
Females ≥40 years	**0.57** [0.21-0.74]***
***Marital status:***	
Married	Reference
Not married	**1.67** [1.17-2.39] ***
Divorced/widowed	0.99 [0.24-4.16]
***Household monthly income (Baht)*:**	
≤10000	1.19 [0.41-3.50]
10001-20000	1.45 [0.71-2.98]
20001-30000	1.44 [0.84-2.49]
>30000	Reference
***Residence:***	
Rural	Reference
Urban	0.89 [0.66-1.20]
***Paid work*:**	
≤10 hours per week	Reference
10-40 hours per week	0.83 [0.51-1.35]
>40 hours per week	0.74 [0.34-1.61]
**Health-risk attributes**	
***Smoking:***	
Never	Reference
Current	**2.45** [1.61-3.74]***
Former	1.09 [0.71-1.66]
***Alcohol drinking:***	
None per week	
1-2 sessions per week	0.72 [0.43-1.20]
3+ sessions per week	1.27 [0.42-3.90]
***Physical activities:***	
None per week	Reference
1-2 times per week	0.68 [0.45-1.03]
3-5 times per week	0.96 [0.65-1.44]
>5 times per week	0.72 [0.39-1.32]
**Health and social attributes**	
***Self-assessed health:***	
Excellent/very good	Reference
Fair	0.97 [0.70-1.34]
Poor or very poor	1.31 [0.74-2.31]
***Cardio-metabolic conditions:***	
No	Reference
Yes	1.33 [0.97-1.84]
***Social interaction:***	
Every week	Reference
1–2 times/month	1.08 [0.57-2.05]
Rarely/never	1.69 [0.62-4.58]

*The Odds Ratio for each variable is mutually adjusted for the influence of all the other variables in the Table.

**The question asks “in the past four weeks, how much of the time did you feel happy?”.

***Statistical significance at p<0.05.

Table 1 presents a breakdown of cohort attributes by unhappiness and mortality status. Approximately 4-5% of cohort members reported being unhappy in 2009 across age-sex categories among those who remained alive four years later. The corresponding percentages were much higher among those who died (4.7-10.7%). Age-sex adjusted bivariate associations linking cohort attributes to unhappiness are also reported in Table 1. Compared to those married, cohort members who were not married or divorced/widowed were 1.35 and 2.34 times more likely to report being unhappy. Former smokers and current smokers were more likely to report being unhappy compared to non-smokers (age-sex adjusted odds ratios 1.22 and 1.89). Alcohol drinking was associated with unhappiness and physical activity was a statistically significant protective factor. Unhappiness was also associated with low social interaction and with self-assessed ‘poor’ or ‘very poor’ health.

Cohort members who reported being happy ‘little’ or ‘none of the time’ in 2009 were more likely to die in the subsequent four years (Adjusted Odds Ratio – AOR 2.60, 95% CI 1.17-5.80). Other significant covariates include being female (<40 years AOR 0.66, ≥40 years AOR 0.57), unmarried (AOR 1.64) and current smokers (AOR 2.45).

Previous reports from Finland suggest possible pathways between unhappiness and mortality. One route is via unintentional injury (hazard ratio 2.83 over 15 years) mediated through adverse health behaviors [8]; an alternative route involves intentional injury (hazard ratio 7.01 over 20 years) through self-harm and suicide [12]. Another report using German socio-economic panel data found that a 10% increase in happiness associated with 4% decrease in probability of dying. The results were more pronounced among males, those unmarried, and the chronically ill [13]. For our Thai Cohort Study, after adjusting for covariates, we also found males and those who never married in 2009 were significantly more likely to die over the next four years. Initial health states (self-assessed overall health and cardio-metabolic conditions) were in the model but they were not statistically significant in the multivariate analyses.

We also investigate how self-reported happiness connects to other standard measures within our cohort. First, in our earlier paper drawing from the same 2009 cohort data, we noted that self-reported happiness correlated with standard measures of life satisfaction [16]. Second, a 2005-2009 longitudinal analysis of low life satisfaction in the cohort (data not shown) confirms that cohort participants who reported being unhappy were much more likely to report low life satisfaction not only in 2009 but also four years earlier (2005). This link to low life satisfaction is important as several studies have shown that low life satisfaction is on the pathway to all-cause mortality [7-9,17,18]. These several strands of evidence collectively indicate that our happiness data have construct and content validity, connecting well to previous literature.

A general relationship between happiness and mortality might seem obvious but there is almost no actual evidence that this is so beyond several reports from affluent economies. Our study provides longitudinal evidence from Thailand and thus is one of the first from a non-affluent region. Our study was prospective and we controlled for a wide array of potential covariates including age, sex, income, behaviours, and health states. Additional variables such as doctor visits were used in other happiness-mortality studies [13] but were not available in our data. Some residual confounding is possible but we note that most of the variables that have been shown to relate to all-cause mortality are in our model. We also acknowledge the limitation of the short mortality follow-up (4 years) and future longitudinal analyses will confirm if the findings hold in the longer term. Even with the small number of deaths investigated, we were able to capture an epidemiologically and statistically significant relationship between self-reported unhappiness and subsequent death.

We conclude that happiness is not just a factor linked to longevity in affluent countries, but also increases the probability of staying alive in a middle-income Asian setting. It would be helpful if this report stimulates studies of happiness and mortality elsewhere in the world. So far it appears the happiness effect noted here is indeed independent of wealth and culture and we expect that this can be found in other settings. The implications are that the pursuit of happiness is likely to add to life expectancy and is a legitimate strategy to improve population health.

### Ethical approval

Informed written consent was obtained from all participants. All students were advised that they could withdraw, or not participate, without any effect on their academic progress. The questionnaires never sought sensitive personal information and no biological samples were taken. Ethics approval was obtained from Sukhothai Thammathirat Open University Research and Development Institute (protocol 0522/10) and the Australian National University Human Research Ethics Committee (protocols 2004/344 and 2009/570).

## Abbreviations

AOR: Adjusted odds ratio; 95% CI: 95% confidence interval; ID: Identity.

## Competing interests

The authors declare that they have no competing interest.

## Authors’ contributions

VY conceptualised the study, analysed data, and wrote a draft manuscript. AS provided comments on original manuscript and revision. AS and SS led the Thai Cohort Study. All authors read and approved the final manuscript.
